# Subdural Empyema as a Complication of Sinusitis: A Diagnosis to Keep in Mind

**DOI:** 10.7759/cureus.53249

**Published:** 2024-01-30

**Authors:** Rosário Eça, Armando Graça, Rita Francisco, Jaime Pamplona

**Affiliations:** 1 Internal Medicine, Centro Hospitalar de Lisboa Central, Lisboa, PRT; 2 Critical Care, Centro Hospitalar de Lisboa Central, Lisboa, PRT; 3 Radiology, Centro Hospitalar de Lisboa Central, Lisboa, PRT

**Keywords:** cross-sectional imaging, neurologic deficit, sinusitis, subdural empyema, intracranial infections

## Abstract

Subdural empyema (SDE) is a rare form of intracranial infection associated with a high morbidity and mortality rate. Infections of the middle ear and paranasal sinuses are the most common predisposing factors that can lead to bacterial proliferation in the subdural space, usually by direct extension in young patients. Clinicians must have a high level of suspicion for patients presenting with concomitant neurological deficits and signs of sinus pathology. Cross-sectional imaging is mandatory for the diagnosis, preferably contrast-enhanced magnetic resonance imaging. Treatment requires a prolonged course of intravenous antibiotherapy and prompt neurosurgical drainage intervention.

Here, we present the case of a 20-year-old patient with long-term neurological sequelae following a left paranasal infection complicated by an SDE. This case report highlights the rapid progression and devastating consequences of SDE, an ominous neurosurgical emergency.

## Introduction

Subdural empyema (SDE) is a rare acute neurological condition accounting for 20% of localized intracranial infections, mostly occurring as a life-threatening complication of sinusitis, otitis, mastoiditis, trauma, or surgical interventions [1]. It represents a loculated pyogenic infection, usually unilateral, located between the dura mater and the arachnoid that can potentially spread over the convexity of the brain and the midline to the contralateral hemisphere [2]. SDE is more common in the second decade of life and in males (male-to-female ratio of 3:1), although incidence has been decreasing over the last decades due to the use of effective antibiotics [2,3].

SDE is a challenging clinical diagnosis because of its non-specific presentation and rapid progression. The symptoms are reflective of increased intracranial pressure, meningeal irritation, and cerebritis, with the most common being fever, headache, vomiting, altered mental status, and focal neurological deficits [3,4]. Time is of the essence, with early treatment increasing the chances of a better outcome, usually requiring both medical and surgical treatment. Although early aggressive treatment of SDE empyema has reduced the mortality rate, it can be associated with significant long-term neurological morbidity [1,5].

## Case presentation

A 20-year-old male was admitted to our emergency department with a five-day history of frontal headache and fever, associated with nausea, vomiting, and photophobia since the previous day. He had a history of uncomplicated sinus disease, and his immunizations were up to date. He lived at home with his parents, none of whom were ill.

At admission, the physical examination revealed a lethargic but arousable patient, oriented to person but not to space or time. The patient had a fever (38.3°C), and his vital signs were stable. His neurologic examination was positive for mild right hemiparesis (left strength and reflexes were normal). The remainder of his physical examination was otherwise normal, and there were no signs of nuchal rigidity. Laboratory workup was remarkable for mild leukocytosis and elevated C-reactive protein. Urgent non-contrast head computed tomography (CT) was relevant for left pansinusitis with left frontal and parafalcine SDE causing a slight contralateral mass effect. A subsequent lumbar puncture showed an elevated white blood cell count of 214 per mm^3^ (with 60% neutrophils) with no microorganisms isolated.

**Figure 1:**
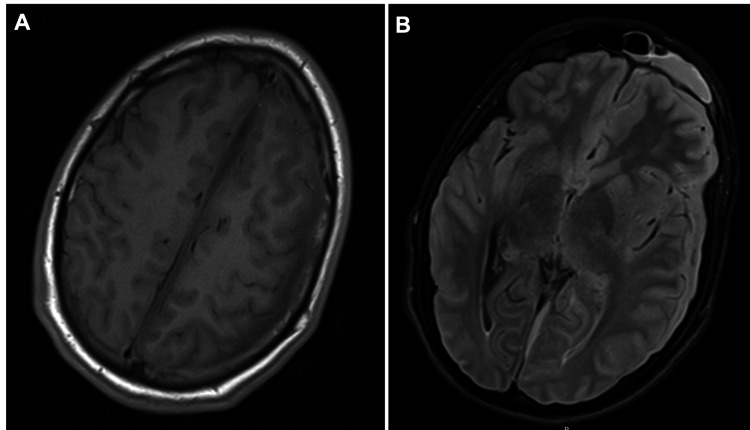
Non-contrast-enhanced cranial MRI at admission. (A) Axial T1 with a subtle extra-axial left parafalcine subdural empyema. (B) Axial FLAIR with an air-fluid level on the left frontal sinus consistent with acute sinusitis. Notice the left frontal subdural empyema causing a slight anterior midline shift. FLAIR: fluid-attenuated inversion recovery; MRI: magnetic resonance imaging

On the following day, his condition significantly worsened with gradually deteriorating mental status to a semi-comatose state and seizures. Afterward, he was admitted to the intensive care unit (ICU), and non-contrast-enhanced magnetic resonance imaging (MRI) confirmed the presence of an SDE with a slight mass effect (Figure 1).

After neurosurgical consultation, an emergent left frontotemporoparietal craniectomy was performed with the evacuation of a large amount of purulent material. Pus culture isolated a *Streptococcus intermedius* and *Enterococcus faecalis* infection and metronidazole was added to the already empirically introduced broad-spectrum antibiotics (ceftriaxone).

**Figure 2:**
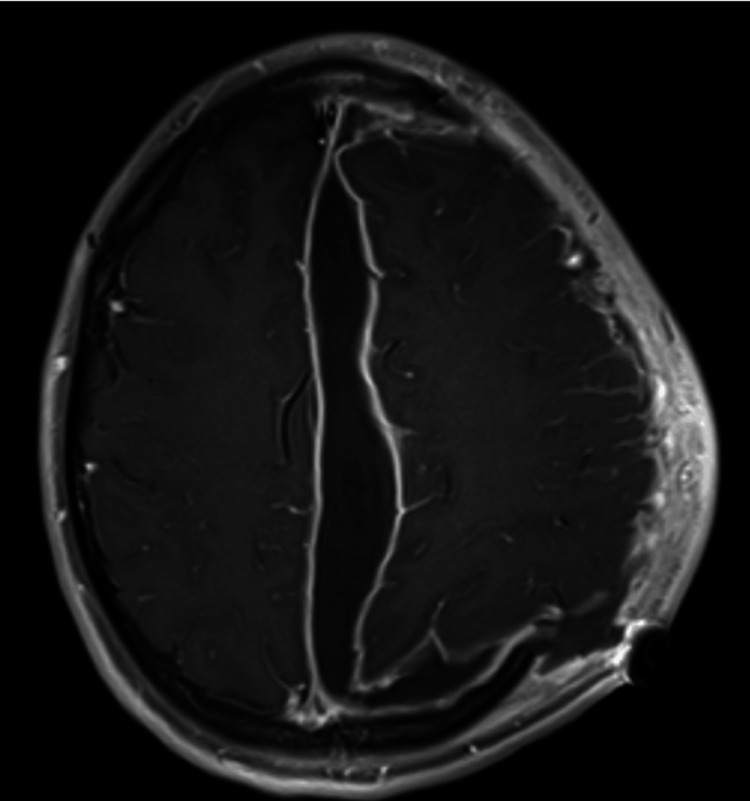
Contrast-enhanced cranial MRI three weeks post-craniectomy. T1 fat-saturated image after intravenous contrast showing a large midline extra-axial fluid collection consistent with relapsed subdural empyema. MRI: magnetic resonance imaging

During the next two weeks in the ICU, the patient’s condition slowly recovered, and follow-up imaging showed a favorable progression. In the third week post-admission, the patient’s clinical condition relapsed with fever and rising inflammatory laboratory parameters; urgent contrast-enhanced MRI was consistent with relapse of the SDE with concerning findings for frontal bone osteomyelitis (Figure 2).

The patient was taken to the operating room for a repeated evacuation of the abscess. No changes were made to the antibiotic regimen being administrated (ceftriaxone plus metronidazole). Afterward, his condition improved, and two months later, he was transferred to the rehabilitation medical floor for continued care.

**Figure 3:**
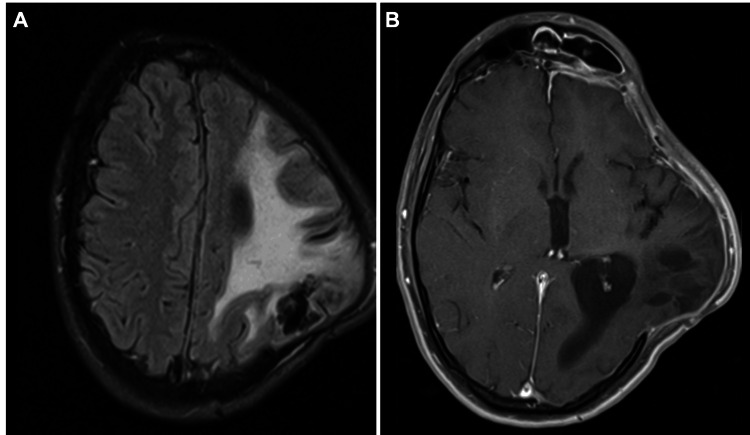
Contrast-enhanced cranial MRI six months post-admission. (A) Axial FLAIR with substantial cortico-subcortical encephalomalacia causing a slight ipsilateral midline shift. (B) Axial T1 fat-saturated contrast-enhanced image with no signs of subdural empyema relapse. Passive left ventricle enlargement (occipital horn) and chronic left frontal sinusitis can be noted. FLAIR: fluid-attenuated inversion recovery; MRI: magnetic resonance imaging

At the time of discharge (six months post-admission), the patient exhibited persistent mild right-sided hemiparesis and expressive aphasia, and the pre-discharge MRI revealed extensive left cortico-subcortical encephalomalacia with stable brain herniation through the craniectomy defect and passive left ventricle enlargement (Figure 3). There was no clinical improvement of the neurological sequelae at the clinical evaluation eight months post-discharge.

## Discussion

Although most cases of sinusitis have a mild and uncomplicated natural history, rarely intracranial complications such as meningitis, empyema, brain abscess, or osteomyelitis can occur [3,4]. It is estimated that the risk of developing suppurative intracranial infection derived from sinusitis or otogenic infections is <0.1% [3]. SDE secondary to sinus pathology can present as an insidious process, with the frontal sinus most frequently implicated, whether by direct extension or retrograde thrombophlebitis via the valveless diploe veins [3,4]. A history of sinus disease is not mandatory as noted by Germiller et al. [6]. In a study of 25 children and adolescents with intracranial complications of sinus disease, only four had a significant history of sinus disease. Germiller et al. [6] also noted that most patients (59%) had a normal neurological examination at presentation and that neurologic deficits at presentation have prognostic significance [6].

Early symptoms of SDE are usually non-specific characterized by acute febrile illness, accentuated by acute neurological deterioration, and, if left untreated, coma and death. Laboratory workup is also non-specific but can show raised inflammation markers. Cross-sectional imaging has improved the rapid diagnosis of intracranial complications and is a cornerstone of the diagnosis of intracranial complications. Although MRI is more sensitive and accurate, head CT is usually done first because of its wide availability and rapid acquisition time [2]. The classic appearance of SDE on a CT scan is of a thin unilateral crescent fluid density collection over the cerebral convexity or along the falx with a rim of contrast enhancement. Of note, SDE exerts a mass effect that can be disproportionate to its volume due to accompanying cerebral edema and ischemia [1,3].

SDE secondary to paranasal sinus infection is commonly polymicrobial, more frequently by anaerobic and microaerophilic streptococci, in particular, those of the *Streptococcus milleri* group as in our patient (*Streptococcus intermedius*) [1,5,6].

Left untreated, SDE is invariably fatal. Since the introduction of antibiotics, the mortality rate has decreased but is still considerable, of up to 28% of patients [3,5]. Treatment of SDE includes broad-spectrum antibiotics, for example, third-generation cephalosporin, along with metronidazole and vancomycin to provide appropriate coverage against pathogens that commonly colonize the upper respiratory tract [3]. Appropriate antibiotic therapy should be tailored to positive culture results and continued for up to six weeks [3]. In the case of intracranial abscesses, emergent surgical drainage is needed to lower intracranial pressure, allowing for the collection of intraoperative cultures [1-3]. Relapse of the pus collection in the subdural space is a known complication, and CT should be repeated if unexplained neurological deterioration occurs [2,4]. Prognosis is mostly dependent on the preoperative mental status, presence of neurological deficits, and aggressiveness of the early treatment. An unfavorable prognosis is more common in older or comatose patients, and up to half of the patients can remain with permanent residual neurological deficits, particularly in older patients [2]. Neurological deficits were present at presentation in our patient, but, unfortunately, there was some initial mismanagement, which led to a potentially hazardous lumbar puncture and a delayed neurosurgical consultation after imaging findings were consistent with a diagnosis of subdural collection.

## Conclusions

Although paranasal sinusitis usually has a benign course, on rare occasions, it can lead to serious and potentially life-threatening complications such as SDE, which should be ruled out in patients with signs of sinusitis and neurological deficits. It is important to note that a history of previous sinus disease is not invariably present, especially in young patients. Early imaging is critical for accurate diagnosis, with successful treatment requiring a combination of aggressive medical and surgical intervention.

## References

[1] Agrawal A, Timothy J, Pandit L, Shetty L (2007). A review of subdural empyema and its management. Infect Dis Clin Pract.

[2] Fernández-de Thomas RJ, De Jesus O (2023). Subdural Empyema. https://www.ncbi.nlm.nih.gov/books/NBK557829/.

[3] Osborn MK, Steinberg JP (2007). Subdural empyema and other suppurative complications of paranasal sinusitis. Lancet Infect Dis.

[4] Silverberg AL, DiNubile MJ (1985). Subdural empyema and cranial epidural abscess. Med Clin North Am.

[5] Niehaus MT, Krape KN, Quinn SM, Kane BG (2018). Frontal sinusitis complicated by a brain abscess and subdural empyema. Radiol Case Rep.

[6] Germiller JA, Monin DL, Sparano AM, Tom LW (2006). Intracranial complications of sinusitis in children and adolescents and their outcomes. Arch Otolaryngol Head Neck Surg.

